# Recirculation and Residency of T Cells and Tregs: Lessons Learnt in Anacapri

**DOI:** 10.3389/fimmu.2020.00682

**Published:** 2020-05-05

**Authors:** Silvia Piconese, Silvia Campello, Ambra Natalini

**Affiliations:** ^1^Dipartimento di Scienze Cliniche Internistiche, Anestesiologiche e Cardiovascolari, Sapienza Università di Roma, Rome, Italy; ^2^Laboratory Affiliated to Istituto Pasteur Italia – Fondazione Cenci Bolognetti, Rome, Italy; ^3^Department of Biology, University of Rome Tor Vergata, Rome, Italy; ^4^Institute of Molecular Biology and Pathology, National Research Council (CNR), Rome, Italy; ^5^Dipartimento di Medicina Molecolare (DMM), Sapienza Università di Roma, Rome, Italy

**Keywords:** T cells, Tregs, cell migration, cell cycle, recirculation

## Abstract

“Location, location, and location”: according to this mantra, the place where living beings settle has a key impact on the success of their activities; in turn, the living beings can, in many ways, modify their environment. This idea has now become more and more true for T cells. The ability of T cells to recirculate throughout blood or lymph, or to stably reside in certain tissues, turned out to determine immunity to pathogens, and tumors. If location matters also for human beings, the inspiring environment of Capri Island has contributed to the success of the EFIS-EJI Ruggero Ceppellini Advanced School of Immunology focused on “T cell memory,” held in Anacapri from October 12, 2018 to October 15, 2018. In this minireview, we would like to highlight some novel concepts about T cell migration and residency and discuss their implications in relation to recent advances in the field, including the mechanisms regulating compartmentalization and cell cycle entry of T cells during activation, the role of mitochondrial metabolism in T cell movement, and the residency of regulatory T cells.

## Introduction

This minireview is inspired by the EFIS-EJI Ruggero Ceppellini Advanced School of Immunology about “T cell memory” 2018 (1) and will expand in further detail two hot topics discussed during the course: T cell migration and residency.

T cell differentiation and function are strictly related to their distribution within different lymphoid and non-lymphoid compartments. In physiological conditions, naive T cells recirculate through secondary lymphoid organs (SLOs), increasing the opportunity to encounter the antigen. After infection, vaccination, or tumor growth, the draining lymphoid compartments undergo dramatic changes, promoting naive T cells’ interaction with antigen-presenting cells and subsequent T cell activation. Activated T cells undergo a strong proliferation (so-called clonal expansion) and deep changes in their metabolism (2, 3). The process culminates with T cell differentiation and the generation of short-lived effectors and long-lived memory cells (4–6). Effector T cells migrate broadly, reaching the site of infection or tumor growth where they exert their effector functions before dying. Memory cells persist in the body, circulating between blood and lymphoid or non-lymphoid tissues as conventional memory T cells, or residing in peripheral tissues as resident memory T cells (Trm) (7). Trm represent a first-line defense against tissue damage and pathogen invasion (8, 9). However, the functional distinction between Trm and conventional effector/memory T cells needs to be clarified. Moreover, it is now clear that some technical caveats may hinder an appropriate and complete analysis of these cells (10). A better understanding of the immunological and metabolic signals dictating the switch between T cell recirculation and residency is needed. Here, we will focus on some emerging concepts regarding this topic: first, the relation between the cell cycle phase and migration during T cell activation; second, the role of mitochondria relocation for T cell movements and compartmentalization; finally, the features of residency of a well-known tissue-infiltrating T cell population, i.e., the regulatory T cells (Tregs).

## T Cell Recirculation and Cell Cycle

After development in the thymus, naive T cells reach the blood circulation, and continuously circulate between blood and SLOs. This journey is finely regulated by the expression of specific homing molecules. Indeed, the L-selectin CD62L expressed by naive T cells mediates their entry into lymph nodes (LNs) by binding ligands expressed on high endothelial venules (HEVs). This binding overcomes blood shear forces, leading to T cell rolling on HEVs (11). At this stage, the interaction between the CC chemokine ligand 21 (CCL21) expressed on HEVs and the CC chemokine receptor 7 (CCR7) on T cells activates the integrin lymphocyte function-associated antigen 1 (LFA1). Activated LFA1 binds the intracellular adhesion molecule 1 (ICAM-1), mediating T cell arrest on the endothelium. Consequently, T cells migrate across the blood vessels and enter the tissue (12). Once in the LN, naive T cells are guided in the paracortical region, also known as T cell zone. In this area, naive T cells interact with dendritic cells (DCs), scanning for the presence of the cognate antigen. It has been estimated that one DC can be scanned simultaneously by up to 500 naive T cells (13, 14). Migration in this area is regulated by a gradient of chemokines and local factors. The chemokine CCL19, produced within the T cell zone, increases T cell motility and promotes T cell–DC interactions by binding CCR7 on the T cell surface (15). Furthermore, after immunization, naive CD8 T cells upregulate CCR5, which binds CCL3 and CCL4 produced at the site of the CD4 T cell–DC interaction in the immunogen-draining LNs (16).

Hence, migration in the SLOs seems to be not only a stochastic process but rather a finely regulated mechanism which increases the probability of antigen recognition. In the case that this rare event occurs, T cells undergo a series of dramatic changes. Resting naive T cells are activated by the integration of three signals: antigen recognition (signal 1), co-stimulation (signal 2), and cytokines, released at the site of T cell–DC interaction (signal 3) (17). This process culminates with the extensive proliferation of antigen-specific T cells, named clonal expansion. T cell expansion is driven by T cell–DC interaction within specialized niches in SLOs and is controlled by several factors which promote the rapid entry of T cell in the cell cycle (18–20). The final goal of this process is to increase the number of T cells capable of eliminating the antigen. It has been estimated that, in the first week of a typical primary T cell response, CD8 T cells can increase their number to about 100 times or more (21). At this point, deregulation of the cell cycle could deeply affect the ability to develop a proper T cell response. For example, a reduced clonal expansion could lead to a decreased number of effector and memory T cells, with consequent loss of protection. Furthermore, it has been hypothesized that the inability to mount an effective primary T cell response in old age and the vaccination failure occurring in elderly persons could be correlated with defects of T cell clonal expansion (22, 23).

Expanding T cells modulate the expression of homing molecules, preparing themselves to reach the peripheral tissue, the site of antigen entry. Retention in SLOs is controlled by the sphingosine-1-phosphate (S1P) receptor expression on T cells. S1P is a lipid molecule that is more concentrated in the blood and in the lymph than in tissues (24). S1P receptor expression is increased in naive T cells, leading to egress from SLOs. Activated T cells upregulate CD69, which prevents S1P receptor expression, holding T cells in the SLOs until the completion of differentiation into effector cells, which can take a few days (25). Once completely differentiated, effector T cells downregulate CD69, and migrate along the S1P gradient. Effector T cells also downregulate CD62L and express chemokine receptors that guide them to the site of infection (26).

**FIGURE 1:**
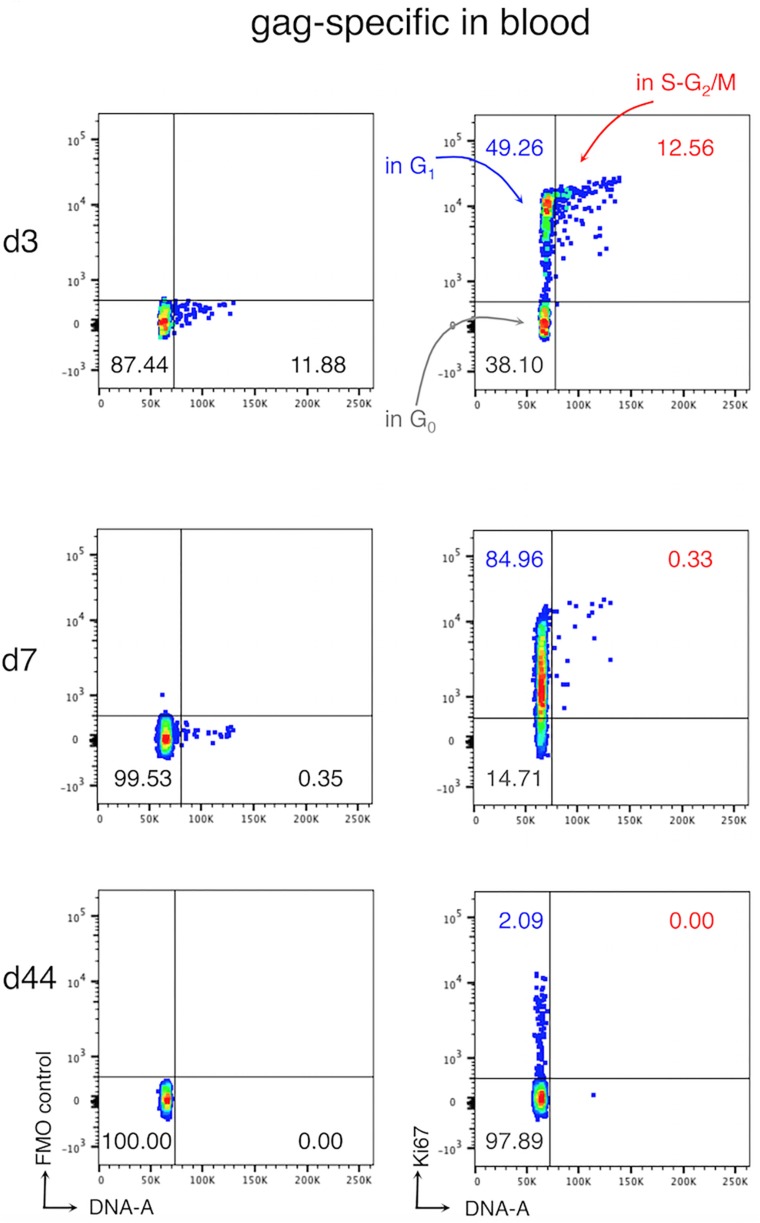
Cell cycle analysis of antigen-specific CD8 T cells in the blood after vaccination. Female Balb/c mice were primed and boosted with viral vectors expressing the model antigen gag of HIV-1. At days (*d*) 3, 7, and 44, post-boost blood was collected and blood cells were analyzed with our new method. The figure shows a typical ki67/DNA staining profile of gag-specific CD8 T cells in the blood. Fluorescence Minus One (*FMO*) controls (*left*) and Ki67 staining (*right*) are shown, as indicated; the *numbers* represent the percentages of cells in the corresponding quadrant Figure adapted from (30).

The kinetic of expansion and migration is poorly defined. Indeed, although it is known that clonal expansion starts in SLOs, the location where activated T cells progress and/or complete their cell cycle is still unclear. To date, the few tools available for the analysis of dividing antigen-specific CD8 T cells, such as cell-labeling dyes and anti-Ki67 antibody, show some important limitations. Indeed, cell-labeling dyes do not allow evaluating whether cells found in one organ proliferated locally or rather migrated in this organ after division (19, 27). Ki67 is a nuclear protein expressed by cells in all the phases of the cell cycle (G1, S, G2, and M), except for those in G0 (or quiescent). Hence, Ki67 analysis alone does not distinguish proliferating cells (in S-G2-M) from those in G1, which may remain for a long time in G1, or even revert to G0 (or quiescent) without dividing (28, 29). We recently set up a new flow cytometric method for the cell cycle analysis of CD8 T cells, which was based on the combination of Ki67 expression and DNA content analyses and allowed us to discriminate between cells in the G0, G1, and S-G2/M phases. By using this method together with a novel gating strategy for the analysis of actively responding T cells, we demonstrated that, at early times after vaccination in mice, cycling antigen-specific CD8 T cells (cells in the S-G2-M phases) were present in the blood, which is usually not considered a site of proliferation (Figure 1) (30). This finding questions the general view by which activated T cells proliferate locally in SLOs and only after completing their cell cycle and differentiation enter the blood circulation, reaching the infection site. In addition, studies on cancer patients have shown that antitumor CD8 T cells increase Ki67 expression after checkpoint inhibitor treatment, suggesting that unleashed T cells can actively cycle in the blood after therapy (31, 32).

## Mitochondrial Dynamics in Memory T Cells and T Cell Migration

In the past, immunologists did not take seriously into account T cell mitochondria since they are poorly represented within a T cell, and T cells are mainly considered as relying on glycolysis for their principal functions. In recent decades, a large body of evidence emerged on the crucial role that the mitochondria, their metabolism, and their morphological dynamics have on these cells. Nowadays, the pivotal role of mitochondrial morphology changes in almost all processes that are essential for a correct T cell development and function is clear and evident (33). Thus, these less attractive organelles suddenly became “main characters” for several immunologists in recent years.

**FIGURE 2:**
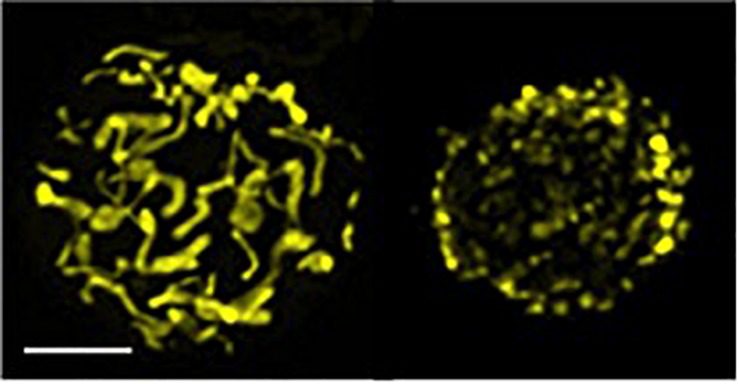
Elongated and fragmented mitochondria morphology in T cells. Confocal z-stack acquisition and 2D reconstruction of an elongated (*left*) or fragmented (*fissed*, *right*) mitochondrial network of Jurkat single cells transfected with mtYFP (*scale bar*, 5 μm). Picture modified from (34).

Mitochondria, the cellular energetic hubs, are highly motile organelles, continuously fusing and fragmenting (a.k.a. fission) their network under the control of the so-called mitochondria-shaping proteins (34) (Figure 2). Drp1 and Dyn2 are the main players controlling fission in concert (35), while mitofusins 1 and 2 and Opa1 are the principal proteins orchestrating mitochondria fusion (36, 37). The balance between these opposing events, at every time or cell demand, determines organelle morphology, which acts as an intracellular signal that instructs different metabolic pathways, reflecting the different physiological functions of the cell. For instance, an elongated network sustains oxidative phosphorylation (OXPHOS) for a correct assembly of the electron transport chain (ETC) complexes, and an optimal ATP production, besides diluting the matrix content (38). A fragmented network, instead, promotes aerobic glycolysis and mitophagy or accelerates cell proliferation in response to nutrient excess and cellular dysfunction (38). Mitochondrial morphology directly regulates T cell differentiation *in vitro* by affecting the engagement of these alternative metabolic routes upon activation. Mitochondrial fusion-dependent fatty acid oxidation with a predominance of OXPHOS is a hallmark of a memory cell signature, while an effector cell subtype mostly relies on fission-dependent glycolysis (39, 40). Thus, mitochondrial dynamics controls T cell fate. Evidence *in vivo* of these findings, together with the molecular mechanisms explaining how mitochondrial dynamics can orchestrate these metabolic shifts and T cell fate, came soon after. Indeed, our lab showed that mitochondrial fragmentation, favoring glycolysis in effector T cells, is dependent on the Erk1-mediated activation of Drp1. Further and interestingly, an additional—but not mutually exclusive—transcriptional mechanism sustains the metabolic shifts in T cell differentiation. Upon T cell receptor (TCR) engagement, in T cells with an elongated mitochondria, the extracellular calcium uptake is exacerbated [presumably because of an inability of the un-fragmented mitochondria to reach the immunological synapse and to buffer calcium (41)], this leading to alterations on the mTOR–cMyc axis, decrease of cMyc expression, and related defective transcription of glycolytic enzymes, cMyc being known as a promoting factor in the transcription of glycolytic enzymes upon T cell activation (42). The consequence is a prominent oxidative metabolism and a memory-like phenotype for these T cells (43). Thus, in sum, memory T cell differentiation is driven by ERK1- and cMyc-dependent mitochondria morphological changes.

More interestingly, for this review’s purpose, the capability of memory T cells to reach the tissues and being resident, rather than to recirculate in the periphery, crucially relies on the ability of these cells to (trans)migrate and extravasate into and from the blood vessels. These basic processes also strictly depend on mitochondrial dynamics. Polarized T cells need to accumulate their mitochondria at the uropod during migration, to fuel the ATP-consuming myosin II cell motor. Drp1-dependent fragmentation of the mitochondria is essential to allow this organelle relocation, while unbalancing the morphology toward an elongated mitochondrial network strongly impairs T cell chemotaxis (44). *In vivo* extravasation and invasion of T cells are regulated likewise. During their trans-migration across an endothelial layer, lymphocytes squeeze and insert their nuclei into a subendothelial pseudopodium (45), a process heavily relying on the activity of the myosin motor (46) and requiring Drp1-dependent mitochondria fragmentation (43). Consistently, *in vivo* Drp1 removal from T cells inhibits their extravasation from the blood toward SLOs, and toward “danger sites” (43).

Noteworthy is that Drp1 knockout (KO) T cells are deficient in cell migration, even though their metabolism is shifted toward an OXPHOS-based metabolism, ideally producing more ATP to fuel the myosin II, which should drive a higher migration rate. This apparent paradox underlines the cell’s need to better modulate the relocation of the mitochondria for a local, subcellular production of mitochondrial ATP rather than for a general mitochondria functionality.

Overall, these findings shed light on a new and crucial role for mitochondrial dynamics in T cell differentiation and function, paving the way for new, and important therapeutic opportunities through pharmacological or genetic manipulation of mitochondria-shaping proteins, also based on memory T cells.

It needs to be considered that forcing mitochondrial fusion during *in vitro* T cell expansion promotes the differentiation of naive T cells toward a memory phenotype, this conferring a higher survival to these cells. However, we observed that T cell migration strictly depends on optimal fragmentation of the mitochondrial network; thus, an unbalance toward mitochondria fusion in memory T cells would inhibit their (trans)migratory capability, therefore impinging on their “choice” to be resident or to recirculate. This observation suggests that a one-way or “chronical” modulation of the activity of mitochondria-shaping proteins could hardly result in successful therapeutic strategies, with this highlighting the actual complexity of the topic. Finally, also in a T cell terminal differentiation into senescence, in which cell migration and proliferation are fatally altered, mitochondria structure, and function result impaired as well (47).

## Tissue Regulatory T Cells: Resident or Recirculating?

Most of the available information about resident T cells come from the study of CD8 Trm, and a growing body of data demonstrates their key role in response to pathogens, in antitumor immunity, in mucosal defense, in vaccine efficacy, and so forth [reviewed in (10)]. Less clear are the identity and functions of CD4 Trm in different contexts, probably because in tissues the CD4 T cell population may comprise variable proportions of Tregs displaying completely different immune functions. Tregs represent a class of CD4 T cells defined by the expression of Foxp3 and exerting non-redundant immunosuppressive and tissue repair functions. In several non-lymphoid tissues, Treg subtypes have been identified that show tissue-specific profiles, differentiate locally in response to variable signals, and perform specialized functions [reviewed in (48)].

Whether tissue Tregs are truly resident cells is still a matter of investigation. Parabiosis experiments have demonstrated that Treg chimerism was lower in the adipose tissue and intestine compared to the spleen, blood, and liver (49–51). When Tregs were further classified into central or effector cells, the latter were found more resistant to recirculation (52, 53); however, this event was transient (52), and upon parabiont disconnection, the chimerism of both effector and central Tregs decayed in a few weeks (52). These results suggest that, at least in certain tissues, effector Tregs may be continuously replenished from circulating Tregs, which locally differentiate and proliferate (54).

When effector Tregs were further subdivided according to the expression of the CD49b integrin, it was possible to distinguish circulating Tregs: indeed, compared to other districts, the blood and highly vascularized tissues (liver and lung) contained a high frequency of CD49b^+^ effector Tregs that displayed a significantly higher rate of exchange between parabiotic mice (55). It could be hypothesized that CD49b^+^ Tregs may be devoted to continuous tissue patrolling through blood circulation, being able to promptly reach damaged or inflamed tissues (55), while the CD49b^–^ cells may show a certain degree of stable residency and exert on-site repair/regenerative functions in physiological settings. For instance, Tregs localize to the epithelial stem cell niche and promote hair growth at the steady state (56). Resident Tregs may exist in the heart protecting from fortuitous inflammation and tissue damage (57). Such tiny and highly specialized Treg populations are settled in locations that are poorly accessible to the circulation and, thus, probably may have acquired better capacities to survive and self-renew locally.

Tregs, or certain Treg subsets, share with Trm some phenotypical markers. For instance, Tregs express CD69 at a higher level in non-lymphoid than in lymphoid tissues (58–60). The expression of CD103 by effector Tregs was established several years ago (61), and CD103^+^ Tregs have been observed at the steady state in several tissues including the lung (58) and the dermis (62). CD39 is a well-recognized marker of Tregs from lymphoid organs (63) and maintained at high levels in tissues like VAT (64). Notably, one of the key transcription factors for the acquisition of a residency program, Blimp1 (65), plays a well-recognized function in the instruction of the effector program in Treg (66). Therefore, in tissues, effector Tregs possess the whole armamentarium that may be needed to establish residency. In this context, a recent paper has shown that the majority of lung-resident CD4 T cells are indeed composed of Tregs that play tissue-protective functions (58).

More elusive is the extent of Treg residency in human tissues. Tregs can be found in several healthy human tissues such as the intestine, skin, adipose tissue, and skeletal muscle (48). In healthy human skin, arginase 2 expression was found as a feature of resident Tregs (67). Whether Tregs can establish long-term residency in these tissues and whether this process may be modified in pathologic conditions remain unclear. Recent analyses in human lung transplant recipients have demonstrated that, contrary to conventional T cells, most Tregs in the bronchoalveolar lavage were of recipient origin (68): this result underscores the dominance of Treg colonization from the blood over persistent Treg residency, at least in this context. According to the mouse data mentioned above (55), it could be suggested that the lung, as a highly vascularized tissue, may be particularly prone to Treg replenishment from the blood and that Treg residency may be more stringent in less vascularized tissues.

The balance between Treg residency and recirculation may have key implications during tissue modifications occurring in chronic inflammation and cancer. Tumor Tregs display a gene signature that combines tissue-specific and tumor-specific genes [reviewed in (69)], and a “core signature” is shared among Tregs infiltrating diverse human cancers (70). In human melanoma, Tregs express a higher level of arginase 2 than in healthy skin (67), suggesting that tumor Tregs may co-opt and enforce signals that preexisted in Tregs resident in the normal parenchyma. In human breast cancer and colon cancer, tumor Tregs were much more similar to the corresponding healthy tissue Tregs than to circulating Tregs (71, 72). However, the analysis of the TCR repertoire of tumor and tissue Tregs led to conflicting results in different tumor types (70–72), and whether tumor Tregs derive from the amplification of Treg clones populating normal tissues, rather than from circulating cells, remains to be ascertained. A deeper understanding of the tumor Treg complexity will be key to designing Treg-targeted therapies that would spare physiological functions of tissue Tregs.

## Discussion

T cell heterogeneity comprises not only a great variety of T cell subpopulations with different functions but also a considerable diversity of migratory patterns. These patterns are strongly related to the function that these cells will exert in a specific tissue. After activation, changes in T cell migratory capacity occur simultaneously with cell expansion and differentiation into effectors and memory cells. Noteworthy is the evidence that cycling antigen-specific T cells are present in the blood in the acute phase of the response, suggesting a very dynamic interplay between cell cycle and migration (30–32). Nevertheless, how clonal expansion and migration are related is still unclear. Interestingly, the elderly show an altered T cell clonal expansion and a worse T cell response to infections and vaccination. However, only a few studies have focused on the possible impact of aging on T cell recirculation (73, 74), and a possible relation is still unclear.

Whether T cells recirculate or reside in one tissue strongly depends on their metabolism: indeed, mitochondrial dynamics regulate T cell migration and differentiation (39, 40, 44). Metabolism could also dictate the survival of certain Trm, i.e., resident Tregs, which exert important tissue homeostatic functions (48). However, in some pathological conditions such as tumors, whether infiltrating Tregs derive from the resident population or are mobilized from the circulating pool remains unclear (70, 72). This review highlights novel concepts of T cell compartmentalization and opens new interesting perspectives regarding the regulation of this process both in physiological and in pathological conditions.

## Author Contributions

SP conceived the review structure. SC prepared the figure. All authors wrote the manuscript.

## Conflict of Interest

The authors declare that the research was conducted in the absence of any commercial or financial relationships that could be construed as a potential conflict of interest.
