# Corrigendum: Recirculation and Residency of T Cells and Tregs: Lessons Learnt in Anacapri

**DOI:** 10.3389/fimmu.2020.01401

**Published:** 2020-07-09

**Authors:** Silvia Piconese, Silvia Campello, Ambra Natalini

**Affiliations:** ^1^Dipartimento di Scienze Cliniche Internistiche, Anestesiologiche e Cardiovascolari, Sapienza Università di Roma, Rome, Italy; ^2^Laboratory Affiliated to Istituto Pasteur Italia – Fondazione Cenci Bolognetti, Rome, Italy; ^3^Department of Biology, University of Rome Tor Vergata, Rome, Italy; ^4^Institute of Molecular Biology and Pathology, National Research Council (CNR), Rome, Italy; ^5^Dipartimento di Medicina Molecolare (DMM), Sapienza Università di Roma, Rome, Italy

**Keywords:** T cells, Tregs, cell migration, cell cycle, recirculation

In the original article, there was a error in the legend of **Figure 1** as published. In the original manuscript we neglected to cite in the legend of **Figure 1** the article from which the figure was taken and adapted. The correct legend appears below.

**Figure 1**. Cell cycle analysis of antigen-specific CD8 T cells in the blood after vaccination. Female Balb/c mice were primed and boosted with viral vectors expressing the model antigen gag of HIV-1. At days (*d*) 3, 7, and 44, post-boost blood was collected and blood cells were analyzed with our new method. The figure shows a typical ki67/DNA staining profile of gag-specific CD8 T cells in the blood. Fluorescence Minus One (FMO) controls (*left*) and Ki67 staining (*right*) are shown, as indicated; the *numbers* represent the percentages of cells in the corresponding quadrant. Figure adapted from (30).

The authors apologize for this error and state that this does not change the scientific conclusions of the article in any way. The original article has been updated.

